# Spike-Triggered Regression for Synaptic Connectivity Reconstruction in Neuronal Networks

**DOI:** 10.3389/fncom.2017.00101

**Published:** 2017-11-08

**Authors:** Yaoyu Zhang, Yanyang Xiao, Douglas Zhou, David Cai

**Affiliations:** ^1^NYUAD Institute, New York University Abu Dhabi, Abu Dhabi, United Arab Emirates; ^2^Courant Institute of Mathematical Sciences and Center for Neural Sciences, New York University, New York, NY, United States; ^3^School of Mathematical Sciences, MOE-LSC and Institute of Natural Sciences, Shanghai Jiao Tong University, Shanghai, China

**Keywords:** spike-triggered regression, network reconstruction, neuronal dynamics, coupling strength inference, inference invariance

## Abstract

How neurons are connected in the brain to perform computation is a key issue in neuroscience. Recently, the development of calcium imaging and multi-electrode array techniques have greatly enhanced our ability to measure the firing activities of neuronal populations at single cell level. Meanwhile, the intracellular recording technique is able to measure subthreshold voltage dynamics of a neuron. Our work addresses the issue of how to combine these measurements to reveal the underlying network structure. We propose the spike-triggered regression (STR) method, which employs both the voltage trace and firing activity of the neuronal population to reconstruct the underlying synaptic connectivity. Our numerical study of the conductance-based integrate-and-fire neuronal network shows that only short data of 20 ~ 100 s is required for an accurate recovery of network topology as well as the corresponding coupling strength. Our method can yield an accurate reconstruction of a large neuronal network even in the case of dense connectivity and nearly synchronous dynamics, which many other network reconstruction methods cannot successfully handle. In addition, we point out that, for sparse networks, the STR method can infer coupling strength between each pair of neurons with high accuracy in the absence of the global information of all other neurons.

## 1. Introduction

Activities of neurons are central to information encoding and processing in the brain. There have been advances in recording neuronal population activity with single cell resolution. For instance, calcium imaging can capture the firing activity of each individual neuron in a population (Stosiek et al., 2003; Grewe et al., 2010). Multielectrode array (MEA) can be deployed to directly measure extracellular signals to obtain spikes of individual neurons in a population through spike sorting (Litke et al., 2004; Field et al., 2010; Shimono and Beggs, 2015). Meanwhile, the intracellular recording can track the membrane potential to reveal the integration of synaptic inputs. Given spike information of a neuronal population obtained from calcium imaging or MEA and the membrane potential traces via intracellular recording of neurons, we ask the question of how to combine these two types of measurement to capture the underlying network structure. In this work, we provide an answer by presenting our spike-triggered regression (STR) method by taking advantage of the common properties of a neuron: (i) the subthreshold dynamics is nearly linear; (ii) the nonlinear suprathreshold dynamics involves a stereotypical spike profile; (iii) neurons interact with one another through spikes. By establishing a linear regression model of the postsynaptic neuron's voltage trace, STR captures statistically the subthreshold voltage response of the postsynaptic neuron triggered by the presynaptic neuronal spikes. In this framework, the regression parameters can describe the dynamical influence between neurons, thus enabling us to reconstruct the underlying connectivity of the network. We demonstrate there is an invariant relation between a regression parameter and the coupling strength for all neuron pairs over different network topologies and dynamical regimes. This allows us to recover coupling strengths between neurons robustly for a neuronal network in general.

To reconstruct neuronal networks, macroscopically, diffusion tensor imaging can be used to study connections across cortical areas but not on the cellular level due to its limited spatial resolution (Le Bihan et al., 2001; Jones and Leemans, 2011). Microscopically, tracing techniques can reveal how a neuron projects its axon to other neurons (Callaway, 2008; Wall et al., 2010), but still limited to a small number of neurons. Meanwhile, many linear statistical methods, such as Granger causality (GC) analysis (Ding et al., 2006), partial directed coherence (Sameshima and Baccala, 1999; Baccala and Sameshima, 2001) and directed transfer function (Kaminski and Blinowska, 1991; Kaminski et al., 1997), have been attempted to tackle the problem of reverse engineering network connectivity from measured activities of neurons. For example, it has been demonstrated that, under certain conditions, the GC connectivity of integrate-and-fire (I&F) neuronal networks coincides with the underlying network topology by using long (~20 min) time series of spike train or voltage trace (Zhou et al., 2013b, 2014). However, since neuronal networks possess nonlinear dynamics, whether these linear-based methods can be in general applied to reconstructing connectivity is yet to be fully addressed. One can in principle employ the information-theoretic measure of transfer entropy, which makes no assumption of the underlying dynamics (Schreiber, 2000; Vicente et al., 2010). Unfortunately, this approach often suffers greatly from the “curse of dimensionality,” i.e., required data length growing exponentially with the network size and the order of memory in time.

To examine the efficiency of our STR method, we use synthetic data generated from conductance-based I&F networks. In contrast to those methods above, our STR only requires relatively short data of 20 ~ 100 s to accurately reconstruct the I&F network topology and the reconstruction also succeeds for nearly synchronous networks or densely connected networks. Furthermore, we show that the accuracy of our STR reconstruction can be improved by increasing data length or sampling rate. Finally, we illustrate an example of recovering the connection to a target neuron using the data type, for example, the voltage trace of the target neuron is measured by intracellular recording and other neurons' spike trains are measured through calcium imaging or MEA.

## 2. Materials and methods

### 2.1. Conductance-based I&F dynamics

In this work, we investigate the network reconstruction of the conductance-based integrate-and-fire (I&F) neuronal network. Experimentally, it has been shown that the I&F models can capture linear subthreshold properties as well as firing statistics of a real neuron (Carandini et al., 1996; Rauch et al., 2003; Burkitt, 2006). Theoretically, the conductance-based I&F neuronal models have been widely applied in large-scale neuronal network modeling and simulations to investigate information processing in various brain areas (Somers et al., 1995; Troyer et al., 1998; McLaughlin et al., 2000; Tao et al., 2004; Cai et al., 2005; Rangan et al., 2005; Zhou et al., 2013a). The I&F neuronal dynamics is governed by the following equation,

(1) $$\begin{array}{r} \frac{\text{d}V^{i}}{\text{d}t}=-G_{\text{L}}\left(V^{i}-\epsilon_{\text{L}}\right)-G_{\text{E}}^{i}\left(V^{i}-\epsilon_{\text{E}}\right)-G_{\text{I}}^{i}\left(V^{i}-\epsilon_{\text{I}}\right), \end{array}$$

where *V*^*i*^ is the membrane potential for the *i*th neuron in the network, $G_{\text{E}}^{i}$ and $G_{\text{I}}^{i}$ are its excitatory and inhibitory conductances, respectively. *G*_L_ is the leakage conductance, ϵ_L_ is the resting potential, ϵ_E_ and ϵ_I_ are the excitatory and inhibitory reversal potentials, respectively. When the membrane potential of a neuron is below the threshold *V*_th_, the neuronal dynamics is described by Equation (1). When the membrane potential of a neuron reaches the threshold *V*_th_, it is reset to the resting potential ϵ_L_ for a refractory period τ_ref_, and evolves again following Equation (1). An action potential of a real neuron is signified by the event of the threshold crossing and voltage resetting in the I&F model. This threshold-reset event is referred to as a firing event (spike) of a neuron. The time evolution of conductances can be explicitly expressed as $G_{\text{E}}^{i}=\underset{j\neq i}{\sum }\underset{k}{\sum }s_{ij}^{+}\alpha \left(\sigma_{\text{GE}},\;\sigma_{\text{HE}},\;t-T_{j,k}\right)+f\underset{l}{\sum }\alpha \left(\sigma_{\text{GE}},\;\sigma_{\text{HE}},t-T_{i,l}^{\text{P}}\right)$ and $G_{\text{I}}^{i}=\underset{j\neq i}{\sum }\underset{k}{\sum }s_{ij}^{-}\alpha \left(\sigma_{\text{GI}},\;\sigma_{\text{HI}},\;t-T_{j,k}\right)$, where *T*_*j,k*_ is the firing time of the *k*th spike of the *j*th neuron, $T_{i,l}^{\text{P}}$ is the arrival time of the *l*th spike of the external Poisson input to the *i*th neuron with strength *f* and rate μ. The alpha function $\alpha \left(\sigma_{\text{d}},\;\sigma_{\text{r}},\;t\right)=\frac{\sigma_{\text{r}}\sigma_{\text{d}}}{\sigma_{\text{r}}-\sigma_{\text{d}}}\left[\exp\left(-t/\sigma_{\text{r}}\right)-\exp\left(-t/\sigma_{\text{d}}\right)\right]\Theta \left(t\right)$, where Θ(*t*) is the Heaviside function, has rising time constant σ_r_ and decay time constant σ_d_. The nonnegative values $s_{ij}^{+}$ and $s_{ij}^{-}$ are the excitatory and inhibitory coupling strengths from neuron *j* to neuron *i*, respectively. For the sake of convenience, we unify the notation of excitatory coupling strength $s_{ij}^{+}$ and the inhibitory coupling strength $s_{ij}^{-}$ into *s*_*ij*_, which is defined as $s_{ij}=s_{ij}^{+}$ if neuron *j* is excitatory and $s_{ij}=-s_{ij}^{-}$ if neuron *j* is inhibitory. Therefore, a positive *s*_*ij*_ indicates an excitatory coupling whereas a negative *s*_*ij*_ an inhibitory coupling.

In our work, we use dimensionless unit for membrane potentials, in particular, *V*_th_ = 1, ϵ_L_ = 0, $\epsilon_{\text{E}}=\frac{14}{3}$, $\epsilon_{\text{I}}=-\frac{2}{3}$. The corresponding unscaled physiological values are *V*_th_ = −55 *mV*, ϵ_L_ = −70 *mV*, ϵ_E_ = 0 *mV*, ϵ_I_ = −80 *mV* (McLaughlin et al., 2000; Cai et al., 2005; Rangan et al., 2005; Zhou et al., 2013a). Time constants retain their dimension with the unit ms. We set τ_ref_ = 2 ms and the conductance time constants σ_GE_ = 2 ms, σ_HE_ = 0.5 ms, σ_GI_ = 5 ms, σ_HI_ = 0.8 ms. Conductance has the unit ms^−1^. In our simulation, $G_{\text{L}}=0.05\;ms^{-1}$, which corresponds to the physiological membrane time constant 20 ms. The numerical method we use to evolve the system (Equation 1) is a fourth order Runge-Kutta method with spike-spike corrections (Rangan and Cai, 2007; Zhou et al., 2009, 2010).

### 2.2. Spike-triggered regression and synaptic connectivity reconstruction

#### 2.2.1. The method

To optimally predict the subthreshold voltage trace of neuron *i* using its own voltage history and the spike train history of other neurons, we establish the following regression model

(2) $$\begin{array}{r} V_{t}^{i}=\beta_{i}^{0}+\overset{p_{1}}{\underset{k=1}{\sum }}\beta_{i}^{k}V_{t-k\tau }^{i}+\underset{j\neq i}{\sum }\overset{p_{2}}{\underset{l=1}{\sum }}\alpha_{ij}^{l}S_{t-l\tau }^{j}+\epsilon_{t}^{i}\text{ for }t\in {𝕁}_{i}, \end{array}$$

for the voltage time series $V_{t}^{i}$ of the *i*th neuron in a network of *N* neurons, where $S_{t}^{j}$ is the binary spike train time series of the *j*th neuron. $S_{t}^{j}=1$ when the *j*th neuron generates a spike in the time interval [*t, t*+τ] and $S_{t}^{j}=0$ otherwise. The sampling time interval length τ is set to a typical value 0.5 ms in the following (See Results for discussion). The parameters *p*_1_ and *p*_2_ are the regression orders of $V_{t}^{i}$ on its own history and on the history of spike trains of other neurons, respectively. The value of *p*_1_ and *p*_2_ can be determined using the Bayesian information criterion (Schwarz, 1978). The complication of nonlinear threshold-reset dynamics is excluded in the linear regression through choosing *t* from the subthreshold region 𝕁_*i*_, such that neuron *i* does not spike or stay refractory in the time interval [*t* − *p*_1_τ, *t*] for *t* ∈ 𝕁_*i*_. Clearly, in all such time intervals [*t* − *p*_1_τ, *t*] with *t* ∈ 𝕁_*i*_, the membrane potential of the *i*th neuron remains in the subthreshold region. Parameters $\beta_{i}^{k}$ and $\alpha_{ij}^{l}$ are determined through the least-square linear regression (Weisberg, 2013). The parameter $\beta_{i}^{k}$ represents the contribution to the prediction of $V_{t}^{i}$ from the subthreshold voltage history of neuron *i* at time lag *k* while the parameter $\alpha_{ij}^{l}$ represents the contribution to the prediction of $V_{t}^{i}$ from the spike train history of neuron *j* at time lag *l*. $\epsilon_{t}^{i}$ is the residual in regression models indicating the prediction error of the *i*th neuron's voltage at time *t* by incorporating the history of the *i*th neuron's own voltage and other neuron's spike trains.

The regression problem of Equation (2) can be explicitly solved from data as follows. By defining ${\text{a}}_{i}\equiv {\left[{\vec{\beta }}_{i},{\vec{\alpha }}_{i1},\cdots \;,{\vec{\alpha }}_{ij},\cdots \;,{\vec{\alpha }}_{iN}\right]}^{T}$, *j*≠*i*, where ${\vec{\beta }}_{i}=\left[\beta_{i}^{0},\cdots \;,\beta_{i}^{p_{1}}\right]$, ${\vec{\alpha }}_{ij}=\left[\alpha_{ij}^{1},\cdots \;,\alpha_{ij}^{p_{2}}\right]$, (·)^*T*^ denotes matrix transpose, and ${\text{x}}_{t}^{i}\equiv {\left[{\vec{V}}_{t}^{i},{\vec{S}}_{t}^{1}\cdots \;,{\vec{S}}_{t}^{j},\cdots \;,{\vec{S}}_{t}^{N}\right]}^{T}$, *j*≠*i*, where ${\vec{V}}_{t}^{i}=\left[1,V_{t-\tau }^{i},\cdots \;,V_{t-p_{1}\tau }^{i}\right]$, ${\vec{S}}_{t}^{j}=\left[S_{t-\tau }^{j},\cdots \;,S_{t-p_{2}\tau }^{j}\right]$, we can cast Equation (2) into the following form

$$V_{t}^{i}={\text{a}}_{i}^{T}{\text{x}}_{t}^{i}+\epsilon_{t}^{i}\text{ for }t\in {𝕁}_{i}.$$

The least-square regression yields ${\text{a}}_{i}=\arg\min\left(\underset{t\in {𝕁}_{i}}{\sum }{\left(V_{t}^{i}-{\text{a}}_{i}^{T}{\text{x}}_{t}^{i}\right)}^{2}\right)$ with an explicit expression for **a**_*i*_

$${\text{a}}_{i}={\left(\frac{1}{n_{i}}\underset{t\in {𝕁}_{i}}{\sum }{\text{x}}_{t}^{i}{\left({\text{x}}_{t}^{i}\right)}^{T}\right)}^{-1}\left(\frac{1}{n_{i}}\underset{t\in {𝕁}_{i}}{\sum }V_{t}^{i}{\text{x}}_{t}^{i}\right),$$

where *n*_*i*_ is the number of discrete time point in 𝕁_*i*_. By the central limit theorem, **a**_*i*_ is asymptotically Gaussian when *n*_*i*_ becomes sufficiently large, i.e., the length of time series becomes sufficiently long. The covariance of **a**_*i*_ can be estimated as

(3) $$\begin{array}{r} cov\left({\text{a}}_{i}\right)={\left(\frac{1}{n_{i}}\underset{i\in {𝕁}_{i}}{\sum }{\text{x}}_{t}^{i}{\left({\text{x}}_{t}^{i}\right)}^{T}\right)}^{-1}\left(\frac{1}{n_{i}\left(n_{i}-1\right)}\underset{i\in {𝕁}_{i}}{\sum }\epsilon_{t}^{i2}{\text{x}}_{t}^{i}{\left({\text{x}}_{t}^{i}\right)}^{T}\right)\\ {\left(\frac{1}{n_{i}}\underset{i\in {𝕁}_{i}}{\sum }{\text{x}}_{t}^{i}{\left({\text{x}}_{t}^{i}\right)}^{T}\right)}^{-1T}, \end{array}$$

which quantifies the uncertainty of the corresponding parameters obtained through regression. By Equation (3), the covariance of regression parameters is proportional to the variance of the regression residual $\epsilon_{t}^{i}$. In general, the source of noise, e.g., the measurement error on voltage or spike timing, can influence the quality of synaptic inference.

#### 2.2.2. Significance test

To reconstruct the connectivity between neurons, we borrow the idea of Granger causality (GC), which identifies causal influence from time series *X*_*t*_ to *Y*_*t*_ if a better prediction of *Y*_*t*_ can be obtained by incorporating the history of *X*_*t*_ (Granger, 1969; Geweke, 1982; Ding et al., 2006). It can be seen from Equation (2) that if $\alpha_{ij}^{l}$ vanishes for all *l* for a certain pair of neuron *i* and neuron *j*, the past information of $S_{t}^{j}$ has no utility in reducing the prediction error $\epsilon_{t}^{i}$ of $V_{t}^{i}$, then neuron *j* does not causally influence neuron *i*. In contrast, if $\alpha_{ij}^{l}\neq 0$ for some *l*, neuron *j* influences neuron *i*. Therefore, we formulate the following null hypothesis

$$H_{0}:\alpha_{ij}^{l}\equiv 0$$

for *l* = 1, 2, ···, *p*_2_. Note that $H_{0}$ is a family of hypotheses, in which $\alpha_{ij}^{l}=0$ holds simultaneously for all *l*. Given a family-wise significance level *r*, we consider the Bonferroni correction (Shaffer, 1995), by which the hypothesis $\alpha_{ij}^{l}=0$ is tested for each *l* simultaneously with significance level *r*/*p*_2_. Then, one can achieve at least 1 − *r* confidence if *H*_0_ is rejected. Note that $\alpha_{ij}^{l}=0$ holds simultaneously for all *l* is equivalent to $\alpha_{ij}^{l_{ij}}=0$, where $l_{ij}=\arg_{l}\max|\alpha_{ij}^{l}/\sigma_{ij}^{l}|$, $\sigma_{ij}^{l}$ is the standard deviation of $\alpha_{ij}^{l}$ estimated through Equation (3) and $\alpha_{ij}^{l}/\sigma_{ij}^{l}$ is asymptotically standard Gaussian. For simplicity of notation, we define $M_{ij}=\alpha_{ij}^{l_{ij}}$. Intuitively, *M*_*ij*_ represents the $\alpha_{ij}^{l}$ that is most likely nonzero over all *l*, thus *M*_*ij*_ = 0 guarantees $\alpha_{ij}^{l}=0$ for all *l*. In the numerical experiments, we observe that *M*_*ij*_ is usually also the peak value of $\alpha_{ij}^{l}$ as a function of *l*. Given significance level *r*, we reject $H_{0}$ if $|{\tilde{M}}_{ij}|>F^{-1}\left(1-r/2p_{2}\right)$, where *F*^−1^(·) is the inverse function of $F\left(x\right)=\frac{1}{\sqrt{2\pi }}\int_{-\infty }^{x}\exp\left(\frac{-t^{2}}{2}\right)\text{d}t$, which is the cumulative distribution function of the standard Gaussian distribution N(0,1) and ${\tilde{M}}_{ij}=M_{ij}/\theta_{ij}$. Here, $\theta_{ij}=\sigma_{ij}^{l_{ij}}$ is the standard deviation of *M*_*ij*_. Otherwise, i.e., $|{\tilde{M}}_{ij}|⩽F^{-1}\left(1-r/2p_{2}\right)$, we accept $H_{0}$. When $H_{0}$ is rejected, we conclude from Equation (2) (as further discussed below) that, for a positive *M*_*ij*_, neuron *i* receives excitatory influence from neuron *j*, whereas, for a negative *M*_*ij*_, neuron *i* receives inhibitory influence from neuron *j*. Note that, by the analysis in section 3.1, $\alpha_{ij}^{l}$ is obtained through regression and can be further interpreted as the subthreshold response kernel triggered by the presynaptic spikes, we term our method as the spike-triggered regression method.

## 3. Results

### 3.1. Inference of coupling strength

It can be seen intuitively from Equation (2) that $\alpha_{ij}^{l}$ encodes the information of how the history of spike train of neuron *j* contributes to the prediction of the subthreshold voltage of neuron *i*. However, it remains an important issue of how $\alpha_{ij}^{l}$ is related to the underlying neuronal dynamics, in particular, the synaptic coupling structure *s*_*ij*_. In the following, we investigate the conductance-based I&F dynamics (Equation 1) to address this issue.

#### 3.1.1. Relation between *M*_*ij*_ and *s*_*ij*_

**Figure 1:**
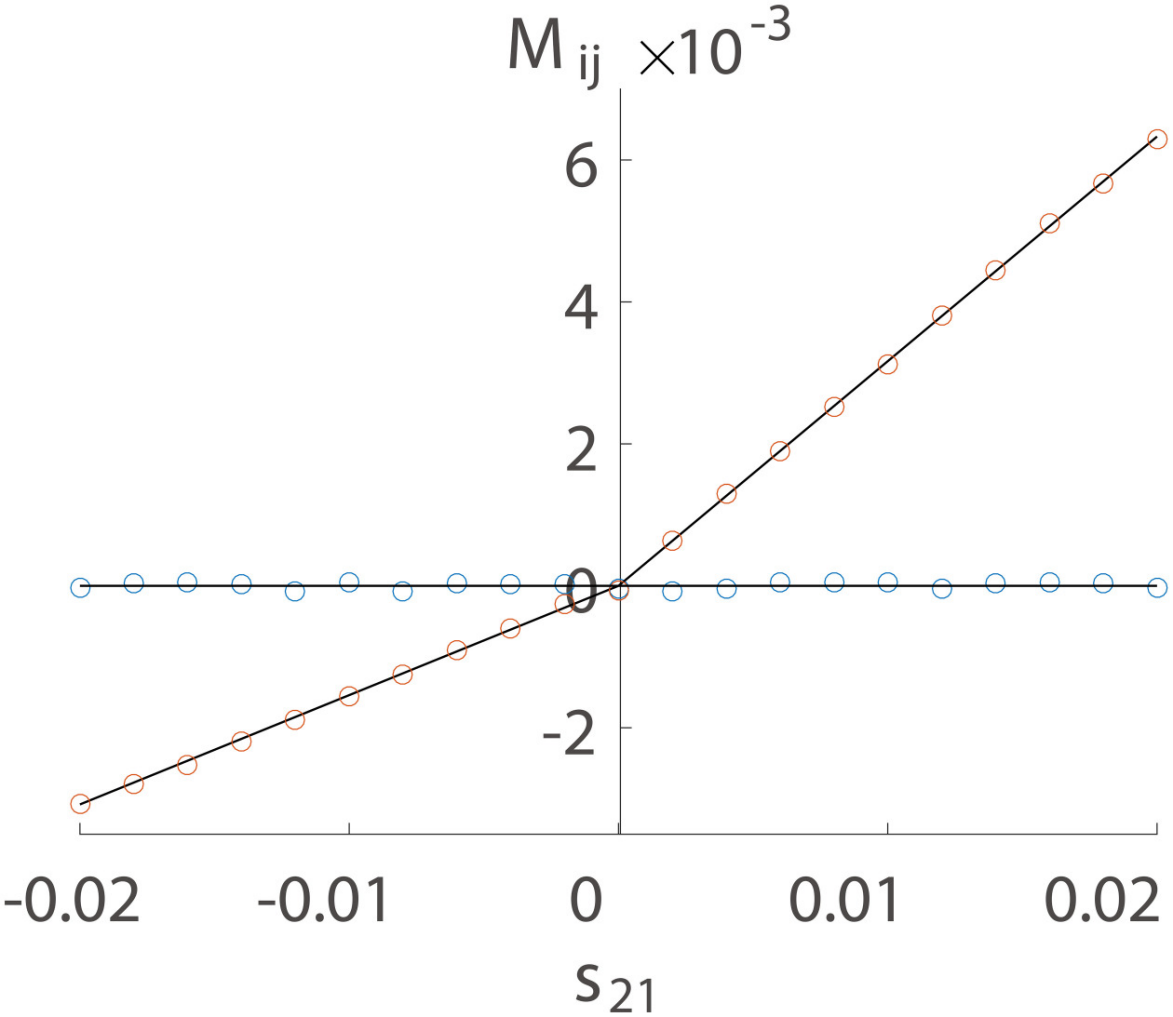
Linear relation between *M*_*ij*_ and *s*_*ij*_. The time series are generated from a unidirectional two-neuron I&F dynamics (Equation 1) with coupling strength *s*_21_ varying from −0.02 to 0.02 and *s*_12_ ≡ 0. Note that a positive *s*_21_ indicates an excitatory coupling whereas a negative *s*_21_ indicates an inhibitory coupling. Each neuron receives independent external Poisson input with strength *f* = 0.012 and rate μ = 1 ms^−1^. Red circles represent the coupled direction from neuron 1 to neuron 2 and blue circles represent the uncoupled direction from neuron 2 to neuron 1. Black lines are a linear fit through the origin for the excitatory and the inhibitory coupling, respectively. The proportionality constants in Equation (4) are determined to be *B*_*E*_ = 0.32 and *B*_*I*_ = −0.15 for the coupled direction.

We first numerically investigate *M*_*ij*_ as a function of *s*_*ij*_ using a two-neuron I&F network with a unidirectional connection from neuron 1 to neuron 2 only. There are two possible situations of connections, excitatory or inhibitory. For either case, we can observe in Figure 1 that *M*_*ij*_ is proportional to *s*_*ij*_ for the excitatory coupling (*s*_*ij*_ > 0) or the inhibitory coupling (*s*_*ij*_ < 0) from neuron 1 to neuron 2. For the uncoupled direction from neuron 2 to neuron 1, *M*_*ij*_ stays nearly 0. This observation suggests the following linear relations:

(4) $$\begin{array}{r} \begin{array}{cc} M_{ij}=B_{E}s_{ij}^{+} & \text{for }s_{ij}=s_{ij}^{+}>0,\\ M_{ij}=B_{I}s_{ij}^{-} & \text{ for }s_{ij}=-s_{ij}^{-}<0, \end{array} \end{array}$$

while *M*_*ij*_ ≡ 0 for uncoupled directions, where *B*_*E*_ is a positive proportionality constant and *B*_*I*_ is a negative proportionality constant. In the following, we use numerical examples to explain this linear relation as well as $\alpha_{ij}^{l}$ in terms of the I&F dynamics.

#### 3.1.2. Response kernel $\alpha_{ij}^{l}$

To understand the relation between *M*_*ij*_ and the coupling strength *s*_*ij*_ of the I&F network, we first rewrite Equation (2) as

(5) $$\begin{array}{r} V_{t}^{i}-\beta_{i}^{0}-\sum_{k=1}^{p_{1}}\beta_{i}^{k}V_{t-k\tau }^{i}=\underset{j\neq i}{\sum }\overset{p_{2}}{\underset{l=1}{\sum }}\alpha_{ij}^{l}S_{t-l\tau }^{j}+\epsilon_{t}^{i}\text{ for }t\in {𝕁}_{i}. \end{array}$$

**Figure 2:**
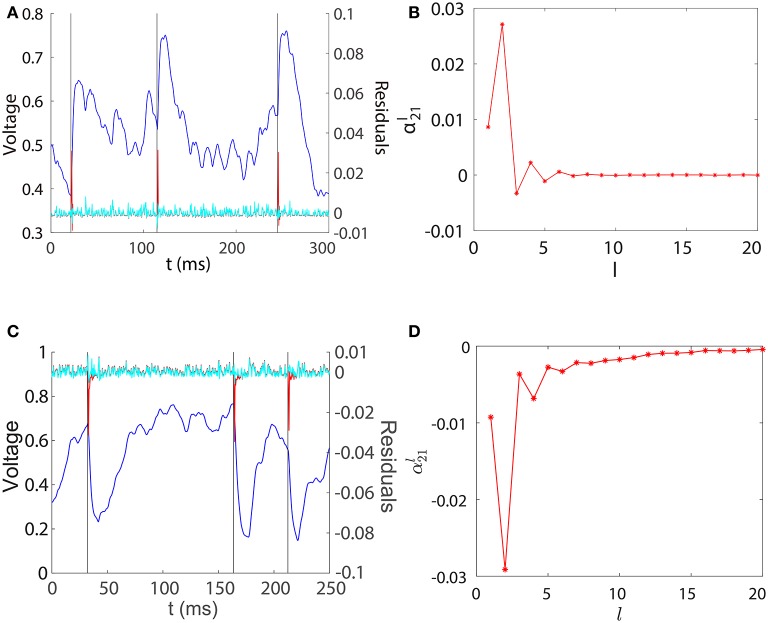
Regression components in STR. **(A)** Spikes of neuron 1 (black), subthreshold voltage trace of neuron 2 (blue), the filtered subthreshold voltage trace $\Delta V_{t}^{\left(2\right)}$ (red), and the regression residual $\epsilon_{t}^{\left(2\right)}$ (cyan) are plotted for the two-neuron I&F network with a unidirectional excitatory connection from neuron 1 to neuron 2 for which *s*_12_ = 0, *s*_21_ = 0.08. **(B)** $\alpha_{21}^{l}$ as a function of *l* obtained from the same network as in **(A)**. **(C,D)** for the case of inhibitory connection. *s*_12_ = 0, *s*_21_ = −0.2. Note that for both excitatory and inhibitory couplings, we have $M_{21}=\alpha_{21}^{\left(2\right)}$ (i.e., *l*_*ij*_ = 2), which attains the peak (absolute) value of $\alpha_{21}^{l}$.

For the convenience of discussion, we denote the left hand side of Equation (5) as $\Delta V_{t}^{i}$, which can be regarded as a filtered subthreshold voltage trace of neuron *i*. Because $S_{t-l\tau }^{j}$ is a binary spike train time series, $\alpha_{ij}^{l}$ as a function of *l* can be interpreted as the linear response kernel of the filtered subthreshold voltage trace $\Delta V_{t}^{i}$ upon receiving a spike from neuron *j*. In Figure 2, we explore the contribution of each regression component to the prediction of $V_{t}^{\left(2\right)}$ and plot the profile of $\alpha_{21}^{l}$ for the unidirectional two-neuron I&F network. For both excitatory and inhibitory coupling, we can observe from Figures 2A,C that $\Delta V_{t}^{\left(2\right)}$ is small, nearly overlapping with $\epsilon_{t}^{\left(2\right)}$, when $V_{t}^{\left(2\right)}$ evolves smoothly within an inter-spike interval of neuron 1. This confirms that the smooth subthreshold dynamics of the I&F neuron can be well captured by the linear prediction of its own history in the absence of spikes from the other neuron.

Figures 2A,C also show that there is a spikelet in $\Delta V_{t}^{\left(2\right)}$ right after a spike of neuron 1. Intuitively, this spikelet structure arises from the rapid change of voltage upon receiving a spike from a presynaptic neuron, and is quantified in the following analysis. Because the subthreshold voltage $V_{t}^{i}$ for neuron *i* evolves smoothly when there is no spike input, it can be well predicted by the linear combination of its own history obtained through regression as follows,

$$V_{t}^{i}\approx \beta_{i}^{0}+\overset{p_{1}}{\underset{k=1}{\sum }}\beta_{i}^{k}V_{t-k\tau }^{i}.$$

When neuron *i* receives a presynaptic spike from neuron *j* (say, excitatory) at time *t*_0_ ∈ [*t*−*mτ, t*−(*m*−1)τ], where *m* is an integer satisfying 1 ⩽ *m* ⩽ *p*_2_, $V_{t}^{i}$ can be expressed as

$$V_{t}^{i}\approx \beta_{i}^{0}+\overset{p_{1}}{\underset{k=1}{\sum }}\beta_{i}^{k}V_{t-k\tau }^{i}+s_{ij}^{+}\left[v_{\text{E}}\left(\Delta t\right)-\overset{m-1}{\underset{l=1}{\sum }}\beta_{i}^{l}v_{\text{E}}\left(\Delta t-l\tau \right)\right],$$

in which Δ*t* = *t* − *t*_0_, *v*_E_(Δ*t*) is the excitatory postsynaptic potential of the unit coupling strength in response to a spike. Therefore, $\Delta V_{t}^{i}\propto s_{ij}^{+}$ in the case of excitation and similarly $\Delta V_{t}^{i}\approx s_{ij}^{-}\left[v_{\text{I}}\left(\Delta t\right)-\overset{m-1}{\underset{l=1}{\sum }}\beta_{i}^{l}v_{\text{I}}\left(\Delta t-l\tau \right)\right]\propto s_{ij}^{-}$ in the case of inhibition. Note that the above analysis depends only on the linearity of the postsynaptic potential response and the form of postsynaptic potentials *v*_E_(Δ*t*) and *v*_I_(Δ*t*) can be general, not limited to dynamics (Equation 1). Subtracting the contribution of $\underset{j\neq i}{\sum }\overset{p_{2}}{\underset{l=1}{\sum }}\alpha_{ij}^{l}S_{t-l\tau }^{j}$ from $\Delta V_{t}^{\left(2\right)}$ yields small residuals (prediction errors) around spikelets as seen in Figures 2A,C. That is, the spikelet structure in $\Delta V_{t}^{i}$ can be quantified by the linear response kernel $\alpha_{ij}^{l}$ (Figures 2B,D). As a consequence, $\alpha_{ij}^{l}$ is linearly related to *s*_*ij*_. This in turn underlies *M*_*ij*_ being linearly proportional to the coupling strength *s*_*ij*_ as exhibited in Figure 1.

### 3.2. Inference invariance

**Figure 3:**
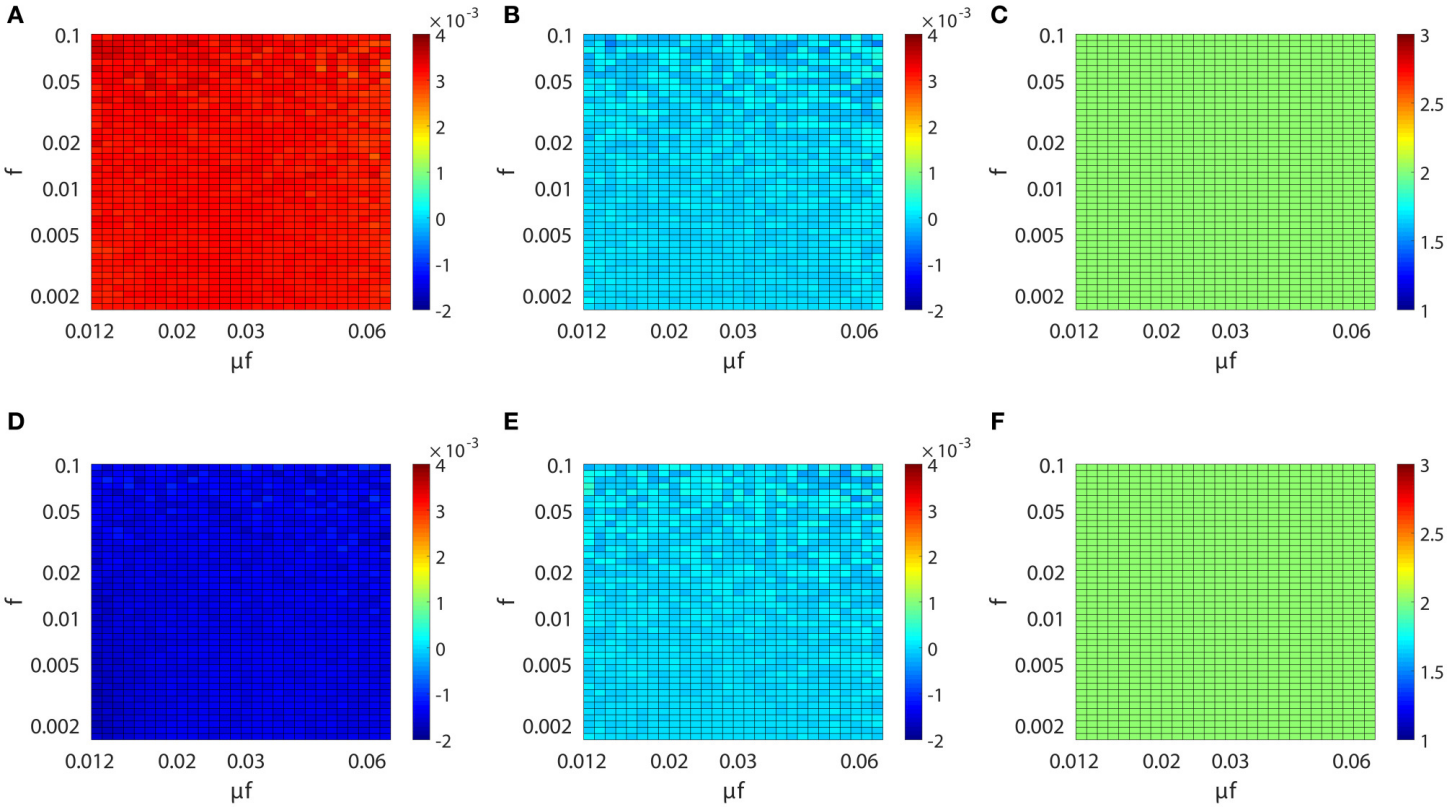
Inference invariance across different dynamical regimes. Upper panel: **(A)** *M*_21_, **(B)** *M*_12_, and **(C)** *l*_21_ as a function of *f* and μ*f* for the excitatory network. Colors code the value of *M*_21_, *M*_12_, and *l*_21_ with the corresponding colorbars. Lower panel: **(D–F)** correspond to the inhibitory case. Both the abscissa and the ordinate are on the log scale in **(A–F)**. The time series are generated from the two-neuron network of I&F dynamics (Equation 1) with a unidirectional coupling *s*_21_ = 0.01, *s*_12_ = 0 for the excitatory case and *s*_21_ = −0.01, *s*_12_ = 0 for the inhibitory case. The Poisson input strength *f* ranges from 0.0016 to 0.1, the Poisson input current μ*f* ranges from 0.012 to 0.062, thus the Poisson input rate μ covers the range from ~0.1 ms^−1^ (100 Hz) to ~40 ms^−1^ (40 kHz) correspondingly.

We have established above the linear relation between *M*_*ij*_ and the coupling strength *s*_*ij*_. Next, we investigate the issue of whether this linear relation is invariant across different dynamical regimes of the neuronal network. Specifically, we study the behavior of the proportionality constants *B*_*E*_ and *B*_*I*_ in Equation (4) for different network input rate μ and strength *f*. Figure 3A (or Figure 3D) displays the result of our scanning of *M*_*ij*_ as a function of *f* and μ*f* for a fixed synaptic coupling *s*_21_ = 0.01 (or *s*_21_ = −0.01), *s*_12_ = 0 for a unidirectional two-neuron network. Note that μ*f* is the mean input current of the Poisson input. In our parameter scan, the Poisson input strength *f* ranges from 0.0016 to 0.1, the Poisson input current μ*f* ranges from 0.012 to 0.062, thus the range of the Poisson input rate μ is from ~0.1 ms^−1^ (100 Hz) to ~40 ms^−1^ (40 kHz) correspondingly. The network dynamics driven by the above Poisson inputs covers a wide variety of dynamical regimes from the highly fluctuating regime to the mean driven regime as firing rate changes from ~1 to 150 Hz.

As observed from Figure 3, for the uncoupled direction, *M*_12_ always remains nearly 0 across different dynamical regimes; whilst for the excitatory (inhibitory) coupling, *M*_21_ is nearly a positive (negative) constant across different dynamical regimes. Therefore, we can conclude that, over broad dynamical regimes, the coupled direction can be well distinguished from the uncoupled direction. It is important to emphasize that *B*_*E*_ (or *B*_*I*_) is approximately a constant over a wide range of dynamical regimes for the excitatory (or inhibitory) coupling between neurons. As will be seen below, *B*_*E*_ (or *B*_*I*_) remains the same for the inference of synaptic coupling between different pairs of neurons in a neuronal network. If the proportionality constant *B*_*E*_ (or *B*_*I*_) is known, we can recover the excitatory (or inhibitory) coupling strength *s*_*ij*_ by *s*_*ij*_ = *M*_*ij*_/*B*_*E*_ (or *s*_*ij*_ = −*M*_*ij*_/*B*_*I*_). In the following, we show that the coupling strengths between neurons obtained from different dynamical regimes can be used to verify the consistency of prediction of the network structure.

For the I&F dynamics (Equation 1), it can be clearly seen that from Figures 3C,F that the time lag *l*_*ij*_ ≡ 2 (i.e., $M_{ij}\equiv \alpha_{ij}^{\left(2\right)}$), which corresponds to the peak of the response kernel $\alpha_{ij}^{l}$, for both excitatorily and inhibitorily coupled directions across different dynamical regimes. This is consistent with what is presented in Figures 2B,D, where *M*_*ij*_ attains the peak (absolute) value of $\alpha_{ij}^{l}$ at *l* = 2 for both excitatory and inhibitory couplings. Therefore, in the following discussion, we will set *l*_*ij*_ ≡ 2 and $H_{0}$ will be rejected when $|{\tilde{M}}_{ij}|>F^{-1}\left(1-r/2\right)$.

### 3.3. A five-neuron network

**Figure 4:**
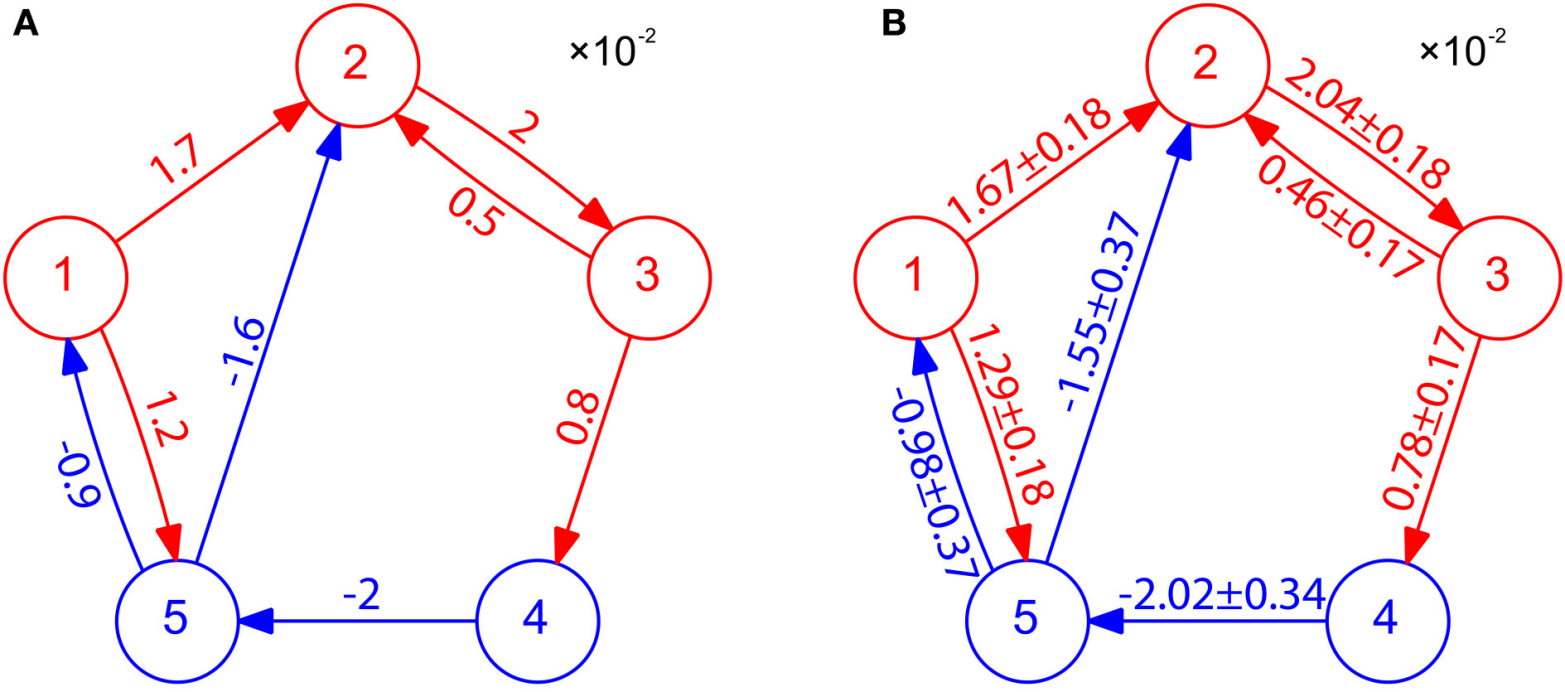
STR reconstruction of an I&F network of 5 neurons. **(A)** Connectivity of the network. Excitatory neurons (red circles) and inhibitory neurons (blue circles) interact with one another through the excitatory couplings (red arrows) and the inhibitory couplings (blue arrows). The coupling strength is labeled on the corresponding arrow with the value of $s_{ij}\times 10^{2}$. Each neuron receives independent external Poisson input with strength *f* = 0.02 and rate μ = 1 ms^−1^. These neurons fire at about 40 Hz. **(B)** Network connectivity reconstructed through STR with significance level *r* = 0.01 using voltage and spike train time series of time length 20 s generated from I&F neuronal dynamics (Equation 1) with the network structure in **(A)**. Invoking the inference invariance with *B*_*E*_ = 0.32 and *B*_*I*_ = −0.15 obtained from the two-neuron network in Figure 1, we label the recovered coupling strength *s*_*ij*_ = *M*_*ij*_/*B*_*E*_ and −*M*_*ij*_/*B*_*I*_ on the predicted excitatory and inhibitory coupling directions, respectively. The predicted values of *s*_*ij*_ with 99% confidence intervals are in good agreement with the true coupling strengths of the underlying network.

We have presented above the case of two-neuron I&F networks to illustrate our method. We now turn to the question of how well our STR method can perform for a multi-neuron network with complex connectivity motifs and different neuron types. Here, we apply our STR method to an I&F network of five neurons consisting of three excitatory neurons and two inhibitory neurons with various coupling strengths (see Figure 4A). Using 20 s of the voltage and spike train time series, we can successfully reconstruct the topological connectivity of the network (see Figure 4B). By the property of inference invariance, using *B*_*E*_ = 0.32 and *B*_*I*_ = −0.15 obtained from the two-neuron network in Figure 1, we can even recover excitatory and inhibitory coupling types and strengths as shown in Figure 4B. The corresponding 99% confidence intervals for the values of couplings are also displayed in Figure 4B. Note that the true values of the coupling strength indicated in Figure 4A all fall within the 99% confidence intervals. In this network there is a type of motif of neuron *X* presynaptically coupled to neuron *Y*, *Y* in turn to neuron *Z*, i.e., *X*→*Y*→*Z* (for example, in Figure 4A, 1 → 2 → 3, 3 → 4 → 5, 4 → 5 → 1 and 4 → 5 → 2 are of this motif). In this motif, *X* does not influence *Z* directly, and it influences *Z* only indirectly through *Y*. As demonstrated in Figure 4, our STR method is able to differentiate the direct influence from the indirect influence and reconstruct the true synaptic connectivity. We emphasize that it takes only 20 s of data for our STR method to provide a rather precise reconstruction of synaptic connectivity with an accurately recovered connectivity strength *s*_*ij*_.

### 3.4. 100-neuron I&F network reconstruction

**Figure 5:**
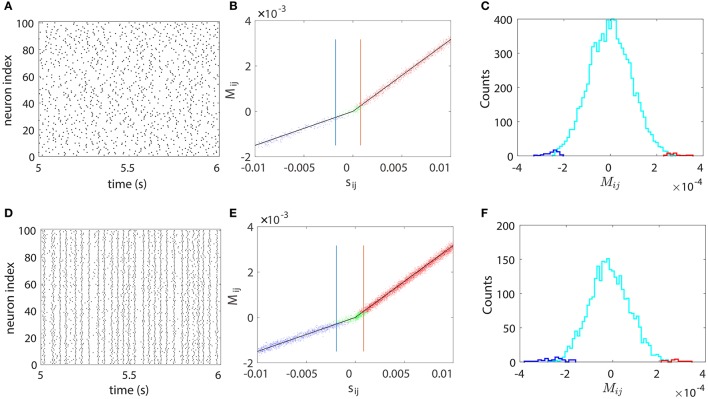
STR reconstruction of a 100-neuron (80 excitatory and 20 inhibitory) I&F network of random connectivity with connection probabilities of 15% (upper panel) and 70% (lower panel). The absolute coupling strength |*s*_*ij*_| of each coupled direction is generated from the uniform distribution *U*(0, 0.01). Each neuron receives independent external Poisson input with strength *f* = 0.012 and rate μ = 1 ms^−1^. **(A,D)** Raster plots of the network firing events, which indicate an asynchronous dynamics in **(A)** and a nearly synchronous dynamics in **(D)**. **(B,E)** *M*_*ij*_ *vs*. the true coupling strength *s*_*ij*_. Each coupled direction is represented by a dot. Shown are directions correctly reconstructed as excitatory (red dots) or inhibitory (blue dots) and the directions incorrectly reconstructed as uncoupled (green dots). Black straight lines indicate the linear relations (Equation 4) between *M*_*ij*_ and *s*_*ij*_ with the proportionality constants *B*_*E*_ = 0.32 and *B*_*I*_ = −0.15 obtained from the two-neuron network in Figure 1. The mean values of θ_*ij*_, which determine the width of the spread of dots around the straight lines, are ~9 × 10^−5^. The blue and red vertical lines indicate critical values $S_{E}^{c}\sim 0.0008$ and $S_{I}^{c}\sim -0.002$, respectively. For all couplings satisfying $s_{ij}>S_{E}^{c}$ or $s_{ij}<S_{I}^{c}$, 99% of them can be correctly predicted. **(C)** Histogram of the predicted *M*_*ij*_ for the uncoupled directions. Shown are directions incorrectly predicted as excitatory (red) or inhibitory (blue) couplings and those correctly predicted as uncoupled (cyan). Here, we use voltage and spike train time series of 100 s with significance level *r* = 0.01. As expected, the area under the cyan curve in **(C)** or **(F)** constitutes ~99% of the uncoupled direction.

We next illustrate our STR method can be successfully used to recover a neuronal network of large size. We apply our STR method to 100-neuron network (80 excitatory neurons and 20 inhibitory neurons) with random topological connectivity of different coupling strengths. The absolute coupling strength |*s*_*ij*_| is generated from the uniform distribution *U*(0, 0.01) to describe the diversity of coupling strength. Figure 5 displays the results for a sparse network and a dense network with connection probability of 15 and 70%, respectively. The sparse network exhibits asynchronous firing activity as in Figure 5A whereas the dense network exhibits nearly synchronous firing activity as shown in Figure 5D. The effectiveness of our STR reconstruction is demonstrated in Figures 5B,C,E,F, respectively, for both networks.

In Figures 5B,E, each connected pair of neurons is represented by a dot which describes the relation between *M*_*ij*_ and *s*_*ij*_. The dots are tightly concentrated around the straight lines of the linear relations (Equation 4) between *M*_*ij*_ and *s*_*ij*_. It should be stressed that, as with the case of the five-neuron network above, the linear relation between *M*_*ij*_ and *s*_*ij*_ with *B*_*E*_ and *B*_*I*_ obtained from the two-neuron network persists and is robustly preserved over different pairs of neurons with different coupling strengths for a large neuronal network. Therefore, the value of *M*_*ij*_ can be used to predict both the coupling type and the coupling strength from neuron *j* to neuron *i*. The width of the spread of dots around the straight lines is determined by the mean value of θ_*ij*_ (standard deviation of *M*_*ij*_) averaged over all directions, which can be used to quantify the uncertainty of reconstruction. In both Figures 5B,E, the mean values of θ_*ij*_ are ~ 9 × 10^−5^. As will be discussed below, the spread width can be reduced by increasing the data length in the STR analysis.

We further note that there are some coupled directions incorrectly predicted as uncoupled (green dots in Figures 5B,E). This arises from the fact that, when the coupling is sufficiently weak, the strength of *M*_*ij*_ can be comparable to or even smaller than its standard deviation, making $H_{0}$ unlikely to be rejected when a finite length of data is used. On the other hand, for a fixed length of time series, when a coupling is sufficiently strong, the value of *M*_*ij*_ can be much larger than its standard deviation, yielding a correct rejection of $H_{0}$. To quantify to which extent a weak coupling can still be successfully predicted, we define excitatory and inhibitory critical values $S_{E}^{c}$ and $S_{I}^{c}$ as follows. First, we reconstruct the topological connectivity of the network with significance level *r* (we usually set *r* = 0.01) using time series of certain fixed duration. Then we compare the predicted and the true connectivity and locate the values of $S_{E}^{c}$ and $S_{I}^{c}$ so that, for all couplings satisfying $s_{ij}>S_{E}^{c}$ or $s_{ij}<S_{I}^{c}$, 99% of them can be correctly predicted. Clearly, the closer the critical value is to 0, the weaker a coupling that we can correctly predict through our STR, hence the more accurate the network reconstruction. In Figures 5B,E, which use 100 s of voltage and spike train times series, the critical values for our STR reconstruction are $S_{I}^{c}\sim -0.002$ for inhibitory couplings and $S_{E}^{c}\sim 0.0008$ for excitatory couplings.

Figures 5C,F displays the histogram of the predicted *M*_*ij*_ for the uncoupled pairs. As expected, the predicted *M*_*ij*_ for the uncoupled directions distributes around 0 and ~99% of the uncoupled directions are correctly predicted, which conforms with the significance level *r* = 0.01. The histogram of *M*_*ij*_ shown in Figures 5C,F is not necessarily Gaussian because each *M*_*ij*_ is from a Gaussian distribution with a different variance.

In Figure 5D, the neuronal network exhibits a nearly synchronous global oscillation of ~35 Hz and the firing pattern of each individual neuron is relatively irregular due to the stochastic Poisson input. In such case, our STR method can still reconstruct the underlying synaptic coupling. Note that the network dynamics shown in Figure 5D is not dominated by inhibition and the firing rate of each neuron is close to the global oscillation frequency. These dynamical features are different from those of the synchronous irregular regime as described in Brunel (2000) where the dynamics are dominated by inhibition and the firing rate of each neuron is much lower than the global oscillation frequency. Furthermore, the dense connectivity also gives rise to a strong effect of indirect influence (i.e., influence mediated by other neurons) between neurons, making it difficult to uncover their true couplings. However, as demonstrated in Figure 5, our STR network reconstruction method performs surprisingly well even for nearly synchronous, densely connected I&F neuronal networks.

Through further examination of many networks with connection probability from 5 to 70%, we can conclude that our STR is able to reconstruct accurately the connectivity of both sparse and dense networks in either asynchronous or nearly synchronous dynamical regime.

### 3.5. STR reconstruction accuracy

**Figure 6:**
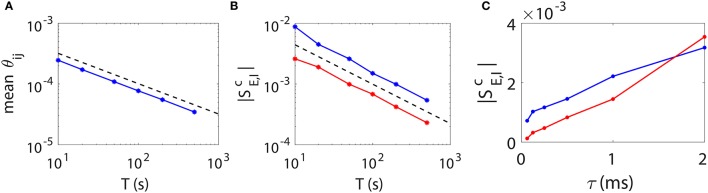
STR reconstruction accuracy as a function of data length *T* and sampling interval length τ. Here, we fix a random I&F network of 100 neurons (80 excitatory and 20 inhibitory) with connection probability 30% and use different data lengths or sampling interval lengths to examine the accuracy of the STR reconstruction. **(A)** θ_*ij*_ averaged over all directions in the network as a function of data length *T*. Black dashed line indicates a $T^{-\frac{1}{2}}$ scaling. **(B)** $\left|S_{E}^{c}\right|$ (red) and $\left|S_{I}^{c}\right|$ (blue) as a function of data length *T*. Black dashed line indicates a *T*^−0.65^ scaling. **(C)** $\left|S_{E}^{c}\right|$ (red) and $\left|S_{I}^{c}\right|$ (blue) as a function of τ. We fix τ = 0.5 ms in **(A,B)** and *T* = 100 s in **(C)**.

As is demonstrated above that our STR method can reconstruct accurately the I&F neuronal synaptic connectivity using time series of 20 ~ 100 s, we further address issues of how one can improve the prediction accuracy. Intuitively, a longer data length should reduce the statistical error and thus improve the network reconstruction accuracy. To determine how long one needs for time series in order to achieve a desired accuracy, we investigate the relation between the STR reconstruction accuracy and the data length *T*. In Figures 6A,B, we present numerical results of two types of quantifications of the network reconstruction accuracy.

First, we consider θ_*ij*_, which represents the statistical uncertainty of *M*_*ij*_. Given a coupled direction with coupling strength *s*_*ij*_ (without loss of generality, we consider *s*_*ij*_ > 0), the mean value of *M*_*ij*_ is approximately *B*_*E*_*s*_*ij*_. It can be easily seen that, the smaller θ_*ij*_, the more likely the hypothesis *M*_*ij*_ = 0 can be correctly rejected. The confidence interval of the predicted coupling strength is determined by θ_*ij*_ as $M_{ij}/B_{E}\pm F^{-1}\left(1/2+c/2\right)\theta_{ij}/B_{E}$, where *c* is the confidence level, *F*^−1^(·) is the inverse function of $F\left(x\right)=\frac{1}{\sqrt{2\pi }}\overset{x}{\underset{-\infty }{\int }}\exp\left(\frac{-t^{2}}{2}\right)\text{d}t$. Clearly, given *c* and *B*_*E*_, the smaller θ_*ij*_ is, the more accurate the predicted coupling strength *M*_*ij*_/*B*_*E*_. To represent the overall accuracy of our synaptic connectivity reconstruction, in Figure 6A, we plot the mean value of θ_*ij*_ averaged over all directions, which controls the width of the spread of dots around the straight lines as in Figures 5B,E, as a function of data length *T*. We can see that it follows a $T^{-\frac{1}{2}}$ scaling as expected from the central limit theorem.

As a second quantification, we use the excitatory and inhibitory critical values $S_{E}^{c}$ and $S_{I}^{c}$ of coupling as the indicator of the accuracy of the network reconstruction. In Figure 6B, both $S_{E}^{c}$ and $S_{I}^{c}$ possess approximately a power law decay as the data length increases. Their decay rate is determined empirically as *T*^−0.65^, which is slightly faster than $T^{-\frac{1}{2}}$. As discussed above, the smaller the critical (absolute) values $S_{E}^{c}$ and $S_{I}^{c}$, the more accurate the weak coupling strengths can be predicted, thus the better network reconstruction.

Based on the above observations, we can conclude that the accuracy of the STR reconstruction follows a scaling of at least $T^{-\frac{1}{2}}$ as the data length *T* increases. Therefore, if one desires to achieve a factor of *Q* (*Q* > 1) improvement in the accuracy of STR reconstruction (i.e., a 1/*Q* times smaller confidence interval, or to detect a 1/*Q* times weaker coupling strength), data of *Q*^2^ times longer length is required.

The accuracy of reconstruction can be further improved through reducing the sampling interval length τ, i.e., increasing the sampling rate. Intuitively, as one samples at a higher rate, i.e., a smaller τ, more information of detailed dynamics is preserved, hence more accurate the network reconstruction. This is confirmed in Figure 6C. Note that a change of the τ value leads to a change of the values of *B*_*E*_ and *B*_*I*_ in the linear relation (Equation 4) between *M*_*ij*_ and *s*_*ij*_. The underlying reason is as follows. As τ vanishes, with the self-prediction residual $\Delta V_{t}^{i}$ decreasing for the smooth dynamics, the reduction of the magnitude of its response kernel $\alpha_{ij}^{l}$ follows the same τ scaling. Therefore, *B*_*E*_ and *B*_*I*_ should be recalibrated when a different τ is used in our STR reconstruction. Furthermore, the firing rate of presynaptic neurons could also influence the accuracy of synaptic connectivity reconstruction. The covariance of $\alpha_{ij}^{l}$ is approximately inversely proportional to the covariance of the presynaptic neurons spike trains. Therefore, if the firing rate of presynaptic neurons is too low, one may need to use longer time series to obtain a high signal-to-noise ratio for an accurate recovery of synaptic connectivity.

### 3.6. Detection of synaptic couplings to a target neuron

**Figure 7:**
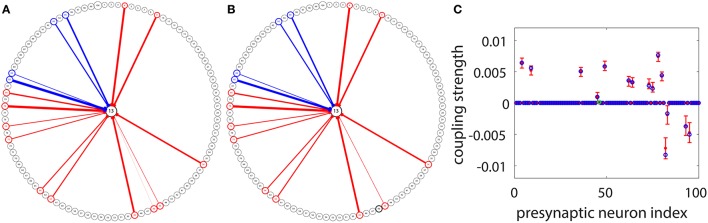
STR reconstruction of synaptic couplings to a target neuron in the I&F network of 100 neurons (Neurons 1 to 80 are excitatory and Neurons 81 to 100 are inhibitory) as in Figure 5A. We use the voltage time series of the target Neuron 13, and the spike trains of the other neurons for the STR reconstruction. **(A)** The underlying synaptic couplings to Neuron 13. **(B)** The predicted synaptic couplings to Neuron 13. The other neurons are arranged on the circle and the couplings among them are not shown. The red and blue arrows indicate excitatory and inhibitory couplings to Neuron 13, respectively. The width of an arrow indicates the strength of the coupling. **(C)** Comparison of the true coupling strengths (blue circles) and the predicted coupling strengths (red dots) to Neuron 13. The 99% confidence interval for each predicted coupled direction is indicated by the error bar around each red dot. We predict the coupling strengths using the proportionality constants *B*_*E*_ = 0.32 and *B*_*I*_ = −0.15 obtained from the two-neuron network in Figure 1. Note that only one coupled direction (from Neuron 46 to Neuron 13) is incorrectly predicted as uncoupled (the green circle in **C**). Its true coupling strength *s*_13,46_ = 0.00017 is much weaker than the excitatory critical value $S_{E}^{c}\sim 0.0008$ shown in Figure 5. Here, we use 100 s of data with significance level *r* = 0.01.

Under the situation where voltage and spike train information of all neurons in the I&F network can be obtained, we have demonstrated the efficiency of our STR method for connectivity reconstruction. However, to apply the STR method to physiological experiments, one may encounter certain constraints from the recording techniques. In experiments, the voltage trace of very few neurons can be acquired through intracellular recording since it is rather difficult to perform intracellular recording on a large set of neurons simultaneously. Nevertheless, we can consider a setup in which the voltage trace of only a target neuron is recorded and the spike trains of many other neurons in the network are obtained through other means, say, calcium imaging (Stosiek et al., 2003; Grewe and Helmchen, 2009; Grewe et al., 2010) or Multielectrode array (MEA) (Litke et al., 2004; Field et al., 2010; Shimono and Beggs, 2015). Applying the STR method to this type of data, we are able to reconstruct the synaptic connectivity from the recorded neuronal population to the target neuron. An example is illustrated in Figure 7. For the same network of 100 neurons as in Figure 5A, we choose Neuron 13 as the target neuron and use our STR method to recover the network couplings to Neuron 13. Note that, we can think of this example as sampling 100 active neurons from a large cortical circuit and the effect of all the unrecorded neurons on each recorded neuron is modeled by an external Poisson input with strength *f* = 0.012 and rate μ = 1 ms^−1^. In general, if spike trains of a much larger neuronal population can be recorded, we could recover these corresponding synaptic connections on the dendritic tree of the target neuron. Here, 100 s of data is used with significance level *r* = 0.01 for reconstruction. Incidentally, we can choose any neuron in the network as a target neuron and obtain a reconstruction similar to what is shown in Figure 7. Comparing Figures 7A,B, we can observe that, except for one very weakly coupled direction (46 → 13), the other couplings to the target neuron are successfully recovered. Displayed in Figures 7C is a detailed comparison between the predicted and the underlying true coupling strengths. Evidently, the underlying true coupling strength falls within the 99% confidence interval of the predicted coupling strength.

## 4. Conclusions and discussion

We have established our STR method and the corresponding significance test for the neuronal network reconstruction. By regressing the subthreshold voltage trace on the spike trains of presynaptic neurons, the subthreshold voltage responses to presynaptic spikes are captured by the response kernel $\alpha_{ij}^{l}$. *M*_*ij*_ has been shown to possess a linear relation with the true coupling strength *s*_*ij*_. Significantly, this linear relation is invariant for any neuron pairs in networks with different coupling structures and over broad dynamical regimes. Therefore, given the proportionality constants *B*_*E*_ and *B*_*I*_, the coupling strength *s*_*ij*_ can be successfully predicted from *M*_*ij*_. It should be emphasized that without the proportionality constants we can still use *M*_*ij*_ as a measure of the relative coupling strength. Our STR method is able to discriminate the direct influence from the indirect influence among neurons and to reconstruct accurately the true synaptic connectivity, even for a nearly synchronous, densely connected network. Often only 20 ~ 100*s* of data is needed for an accurate reconstruction. The accuracy of our STR reconstruction can be further improved by increasing the length of data or the sampling rate. Finally, for potential application in experiments, an example is illustrated for reconstructing the couplings to a target neuron under the setting, where the voltage of the target neuron is measured through intracellular recording and the spike trains of other neurons are obtained through calcium imaging or MEA. In summary, our STR method can efficiently reconstruct the neuronal synaptic connectivity, thereby, provides a means of shedding light on how neuronal networks are organized to perform functions. Note that, it is in general quite challenging in experiment to obtain the true underlying synaptic connectivity of large neuronal networks to verify any theoretical network reconstruction method. We expect to verify our STR method in future collaborations with experimental labs.

**Figure 8:**
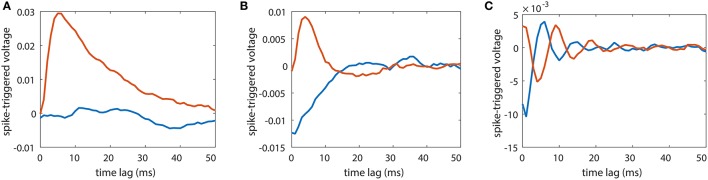
Spike-triggered average (STA) for a unidirectional two-neuron network of I&F dynamics (Equation 1). The coupling strength is fixed to *s*_21_ = 0.01 and *s*_12_ = 0. Red curves indicate STA of the voltage of neuron 2 triggered by the spikes of neuron 1 whereas blue curves indicate the STA of the voltage of neuron 1 triggered by the spikes of neuron 2. The STA results are shown in three different dynamical regimes for external Poisson inputs with strength *f* = 0.04 and rate **(A)** μ = 0.16 ms^−1^, **(B)** μ = 0.3 ms^−1^ and **(C)** μ = 1 ms^−1^. The neurons fire at about **(A)** 1 Hz, **(B)** 15 Hz and **(C)** 100 Hz.

A widely used strategy to infer the synaptic connectivity between neurons is the spike-triggered average (STA) method. It has been applied to reconstruct the excitatory or inhibitory postsynaptic potential in electrophysiological study. It assumes linear response dynamics and aims to capture the response kernel through averaging the trajectory of the postsynaptic neuron's voltage response upon the presynaptic neuron's spikes. In Figure 8, we apply STA to a unidirectional two-neuron network with *s*_*ij*_ = 0.01 over different dynamical regimes as in Figure 3A. It can be seen that, STA results are quite different over different dynamical regimes even if the synaptic coupling remains unchanged. In Figure 8A, when the firing rate of the neuron is about 1 Hz and STA of the coupled direction from neuron 2 to neuron 1, i.e., the voltage response of neuron 1 triggered by the spikes of neuron 2, reflects the underlying synaptic coupling from neuron 2 to neuron 1 while STA of the uncoupled direction from neuron 1 to neuron 2 stays around 0 indicating no synaptic coupling from neuron 1 to neuron 2. However, in Figures 8B,C, STA of the uncoupled direction significantly deviates from zero indicating the existence of synaptic coupling. Moreover, in Figure 8C, STA of the coupled direction oscillates around zero, making it difficult to infer whether the coupling type is excitatory or inhibitory. Therefore, STA in general cannot be used for the synaptic coupling reconstruction in neuronal networks. In contrast, as demonstrated in Figure 3, our STR is robust for the synaptic connectivity reconstruction over a wide range of dynamical regimes.

**Figure 9:**
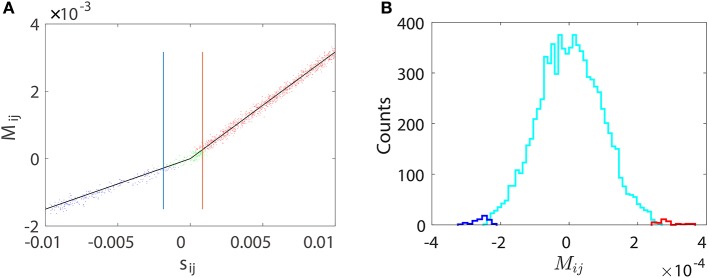
Pairwise STR reconstruction of a random I&F network of 100 neurons (80 excitatory and 20 inhibitory) with sparse connectivity as in Figure 5A. $\alpha_{ij}^{l}$ is obtained using only the voltage trace of neuron *i* and the spike train of neuron *j* by disregarding the activity of all other neurons. **(A)** *M*_*ij*_ vs. the true coupling strength *s*_*ij*_ is represented by a dot for each coupled direction. Shown are directions correctly predicted as excitatory (red dots) or inhibitory (blue dots) couplings and directions incorrectly predicted as uncoupled (green dots). Black lines indicate the linear relations (Equation 4) between *M*_*ij*_ and *s*_*ij*_ with the same values of *B*_*E*_ = 0.32 and *B*_*I*_ = −0.15 as obtained from Figure 1. The width of the spread of dots around the straight lines is controlled by the mean value of θ_*ij*_, which is ~9 × 10^−5^. The critical values $S_{I}^{c}\sim -0.002$ and $S_{E}^{c}\sim 0.0008$ for inhibitory and excitatory couplings, respectively. **(B)** Histogram of *M*_*ij*_ for the uncoupled directions. Shown are directions incorrectly predicted as excitatory (red) or inhibitory (blue) couplings and directions correctly predicted as uncoupled (cyan). Here, we use voltage and spike train time series of 100 s with significance level *r* = 0.01. As expected, the area under the cyan curve in **(B)** constitutes ~99% of the uncoupled direction.

We now address another important issue about the lack of neuronal activity recordings of an entire neuronal circuit. Theoretically, if the spike trains of all neurons in the network are incorporated in the regression, the indirect influence between two neurons mediated by other neurons can be well removed and the direct synaptic coupling can be successfully inferred by STR. However, due to the limitation of recording techniques, one can usually obtain only the spike trains of a subnetwork of neurons in experiments. Under this condition, the indirect influence between two neurons mediated by unmeasured neurons could influence the inference of the direct coupling. To investigate the question of whether our STR method can still provide a reliable reconstruction of the connectivity of a subnetwork, we consider the sparsely connected network as in Figure 5A. We apply our regression method to each pair of neurons by ignoring all other neurons in the network. The results of the pairwise STR method are summarized in Figure 9. In Figure 9A, dots describe *M*_*ij*_ obtained through the pairwise STR as a function of *s*_*ij*_. Importantly, the dots are also narrowly concentrated around the straight lines with an identical proportionality constants (*B*_*E*_ = 0.32 and *B*_*I*_ = −0.15) to what is obtained from the unidirectional two-neuron network in Figure 1. The width of the spread of dots around the straight lines is quantified by the mean value of θ_*ij*_, which is ~9 × 10^−5^ approximately the same as in Figure 5B. The critical values $S_{I}^{c}\sim -0.002$ and $S_{E}^{c}\sim 0.0008$ for inhibitory and excitatory couplings, respectively, are nearly identical to those in Figure 5B. Consistent with the significance level *r* = 0.01, ~99% of the uncoupled directions are correctly predicted. Therefore, for a sparse network, even when we obtain only the activities of a subnetwork of neurons, the pairwise STR method is able to reconstruct reliably the corresponding neuronal couplings of the subnetwork and to achieve approximately the same accuracy as the conditional STR reconstruction, which uses the knowledge of activities of other neurons in the network. Intuitively, when the synaptic connectivity is sparse, for a pair of coupled neurons, the overall indirect influence is mediated by a very small number of other neurons through long, indirect paths, resulting in much weaker effects than the direct one. As a consequence, the underlying synaptic connectivity can be often successfully recovered through the pairwise STR application. As suggested by many studies that the structural brain connectivity may form a sparse graph (Song et al., 2005), our STR method can be potentially applied to subnetwork recordings of a cortical area to reconstruct the subnetwork connectivity.

**Figure 10:**
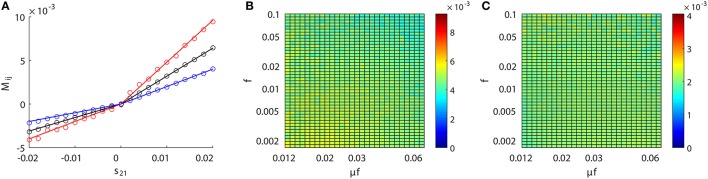
STR network reconstruction with additive noise. Two types of noise are added separately to the data—additive Gaussian white noise with standard deviation of 0.01 in dimensionless unit (See section 2.1 for details) on voltage and spike-jittering with a uniform distribution over the interval [−0.5, 0.5 ms] on presynaptic spike trains. **(A)** Linear relation between *M*_*ij*_ and *s*_*ij*_ for the same unidirectional two-neuron network as in Figure 1 with no measurement noise (black circle), additive noise on voltage (red circle) or spike-jittering (blue circle). The straight lines indicate the corresponding linear relations fitted from the data points. Invariance of *M*_*ij*_ with **(B)** additive noise on voltage or **(C)** spike-jittering over a wide range of dynamical regimes for a unidirectional two-neuron network with *s*_*ij*_ = 0.01 as in Figure 3.

In addition, the presence of noise usually complicates the reconstruction of synaptic connectivity since it gives rise to lower signal-to-noise ratio. In the above neuronal networks, the major source of noise comes from the external Poisson input, which leads to fluctuations in subthreshold voltage and firing dynamics of neurons. However, our STR method is robust to this type of noise as shown in Figure 3. Another source of noise may come from the measurement error in subthreshold voltage and spike timing in experimental recordings. In Figure 10, we investigate the effects of this source of noise on our STR reconstruction: additive Gaussian white noise with standard deviation of 0.01 in dimensionless unit (See section 2.1 for details) on the recorded voltage or spike-jittering with a uniform distribution over the interval [−0.5, 0.5 ms] in recorded spike trains of presynaptic neurons. In Figure 10A, we can observe that the linear relation well holds with these two types of noise added to the measured dynamics of neurons. In Figure 10B, one may notice that *M*_*ij*_ tends to be lower for larger *f* and μ*f*. Therefore, we need to reconstruct the synaptic coupling strength separately over different dynamical regimes in the presence of large measurement noise of voltage. In Figure 10C, *M*_*ij*_ for the coupled direction stays constant over a wide range of dynamical regimes. Therefore, the synaptic connectivity reconstruction in the presence of spike-jittering can be performed similarly as that discussed previously. Note that the proportionality constant varies with different types of noise as shown in Figure 10A, therefore, it should be chosen correctly to recover the underlying synaptic coupling strength. In addition, the quantal release nature of the synaptic transmission may lead to a different postsynaptic potential amplitude for each presynaptic spike. This may be modeled using a different *s*_*ij*_ upon each presynaptic spike. From our analysis in section 3.1, it is expected that only the mean value of *s*_*ij*_ could be recovered from *M*_*ij*_ in our STR method. In experiment, variability in the neuronal dynamics could also complicate the synaptic connectivity reconstruction. Because the subthreshold membrane potential response behavior is usually different for different types of neurons, one may need to recalibrate the proportionality constant for each type of neuronal dynamics for the recovery of synaptic connectivity.

## Author contributions

Conceived the research: YZ. Designed the research, performed experiments, analyzed data and wrote the paper: YZ, YX, DZ, and DC.

### Conflict of interest statement

The authors declare that the research was conducted in the absence of any commercial or financial relationships that could be construed as a potential conflict of interest. The reviewer GM and handling Editor declared their shared affiliation.
